# Idiopathic pseudoaneurysm of the popliteal artery with endovascular treatment: A case report

**DOI:** 10.1016/j.radcr.2023.06.062

**Published:** 2023-07-14

**Authors:** Martin Rief, Angelika Rief, Helmar Bornemann-Cimenti, Peter Rief

**Affiliations:** a Division of Anesthesiology and Intensive Care Medicine, Medical University of Graz, Graz, Austria; b Department of Obstetrics and Gynecology, Medical University of Graz, Graz, Austria; c Clinical Department of Angiology, Medical University of Graz, Graz, Austria

**Keywords:** Popliteal artery, Aneurysm, False, Ultrasonography, Doppler, Duplex, Endovascular aneurysm repair

## Abstract

Popliteal pseudoaneurysms are a rare vascular pathology, usually caused by trauma or iatrogenic interventions. Idiopathic cases are exceptionally uncommon. This case report aims to describe the diagnosis and successful endovascular treatment of an idiopathic pseudoaneurysm of the popliteal artery in a 90-year-old bedridden female patient presented with acute pain and swelling in the left knee at the emergency department. The patient underwent successful endovascular treatment with a covered stent and thrombin injection, leading to complete exclusion of the pseudoaneurysm. Popliteal pseudoaneurysms are a rare pathology, and idiopathic cases are even more uncommon. Endovascular therapy for popliteal pseudoaneurysms is associated with lower morbidity and mortality rates compared to open surgical repair. This case report highlights the importance of interdisciplinary collaboration between vascular surgeons and interventional radiologists in the management of rare vascular pathologies.

## Introduction

Pseudoaneurysms, also known as false aneurysms, are a common complication that arises after injury to the outer layers of an artery. These injuries result in blood flow between the tunica media and tunica adventitia, leading to the development of a hematoma and compression of surrounding tissues, as well as inflammatory processes [1]. While the common femoral artery is the predilection site for pseudoaneurysms due to its frequent use in invasive procedures, pseudoaneurysms of the popliteal artery (PPAs) are rare and can have various causes such as blunt or penetrating trauma, fractures, previous surgery, infections, and osteochondromas [2], [3]–14]. However, idiopathic PPAs are exceedingly uncommon.

This case report aims to describe a minimally invasive treatment approach and noninvasive diagnostic procedures for an idiopathic pseudoaneurysm of the popliteal artery in an elderly, bedridden patient. Therefore, this report serves as an important contribution to the medical community's knowledge of PPAs and their treatment options, especially in the case of an idiopathic PPA where underlying causes are difficult to identify.

## Case presentation

A 90-year-old female patient was admitted to the emergency department with complaints of acute pain and swelling in her left knee. The patient's reduced state of health made it difficult to obtain an accurate case history. According to a written report from her general practitioner, the patient had experienced symptoms for approximately 24 hours before presenting to the hospital, and there was no history of surgery or any type of intervention. Given the patient's bedriddenness and multimorbidity, a history of trauma to the affected limb was not considered feasible. Further diagnostic testing was performed to determine the cause of the patient's symptoms, leading to the diagnosis of an idiopathic pseudoaneurysm of the popliteal artery.

Upon clinical examination, the patient was observed to have an emaciated body with incipient flexion contracture in both knee joints. However, all vital parameters such as heart rate, blood pressure, body temperature, and oxygen saturation were within normal range. Physical examination of the affected limb revealed a pulsating tumor in the left popliteal fossa and moderate swelling of the left lower leg. There were no clinical signs of bone fracture nor critical limb ischemia, such as abnormal temperature, pallor, or cyanosis.

**Fig. 1:**
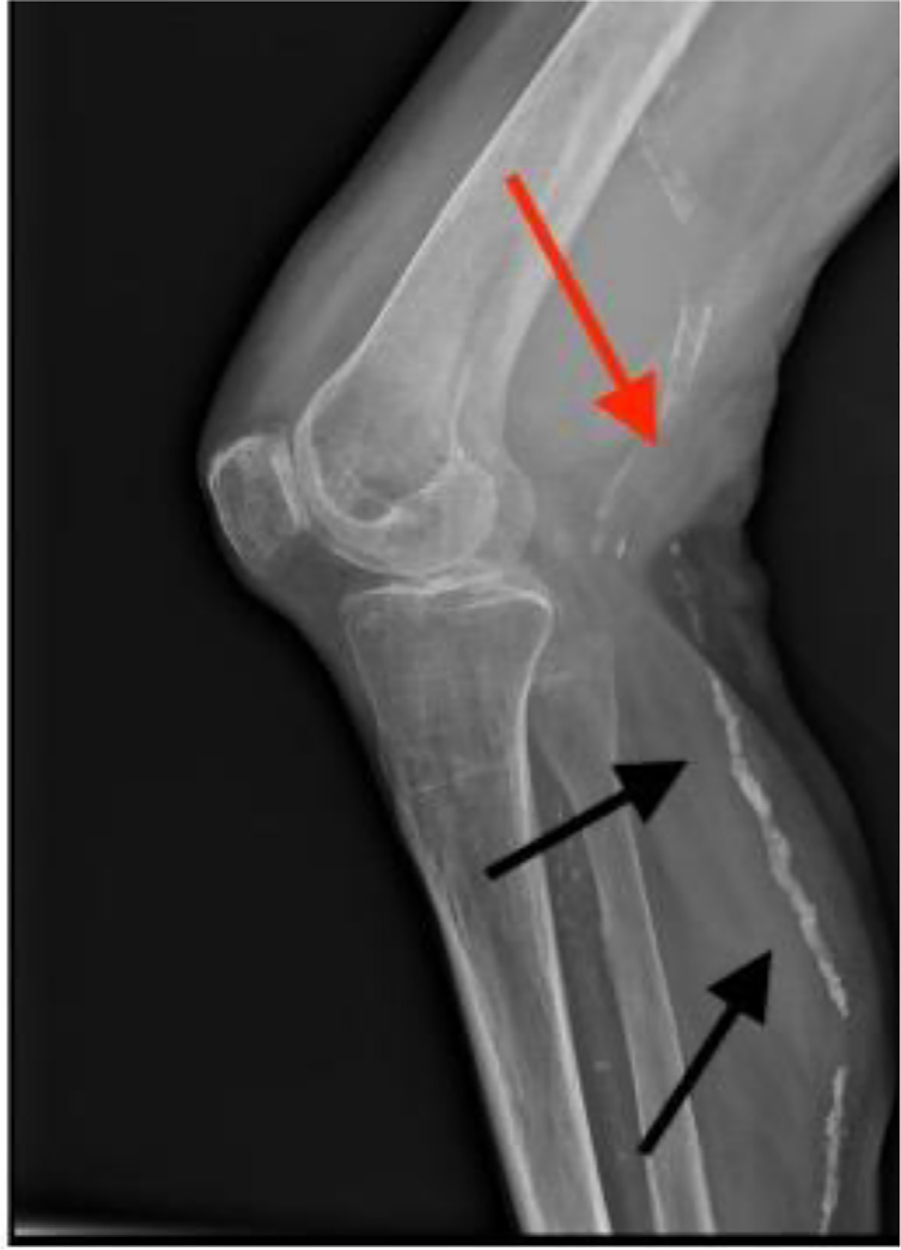
X-ray of the left knee joint, lateral view. Legend 1: Backward arched displacement of the calcified popliteal artery, black arrows indicate the calcified vein and red arrow marks the expansion already visible in the x-ray caused by the pseudoaneurysm of the popliteal artery.

**Fig. 2:**
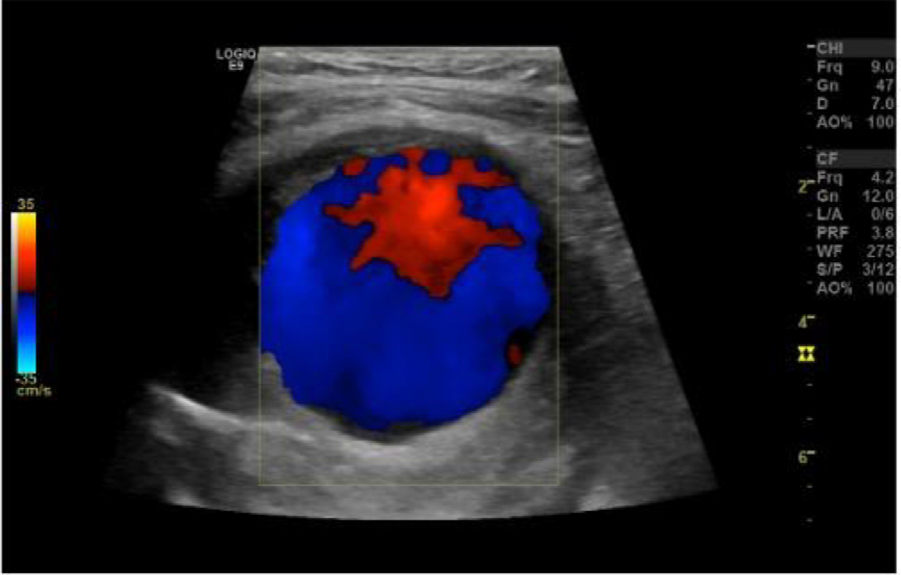
Duplex sonography of the popliteal artery. Legend 2: Duplex ultrasonography shows extraluminal blood flow of the popliteal artery.

**Fig. 3:**
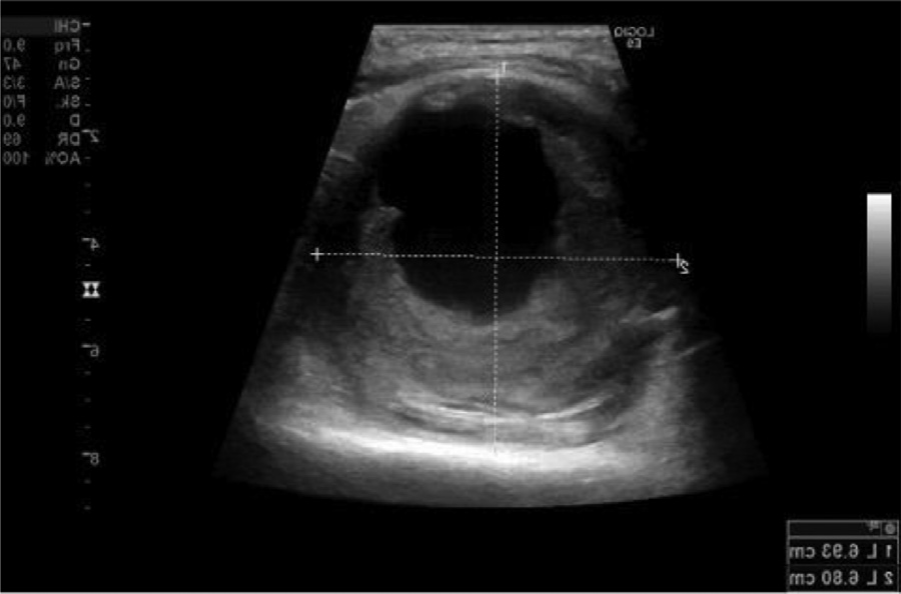
Ultrasonography of the popliteal artery. Legend 3: Hematoma with marginal thrombotic formations, size measurement of the pseudoaneurysm (6.8 × 6 cm).

**Fig. 4:**
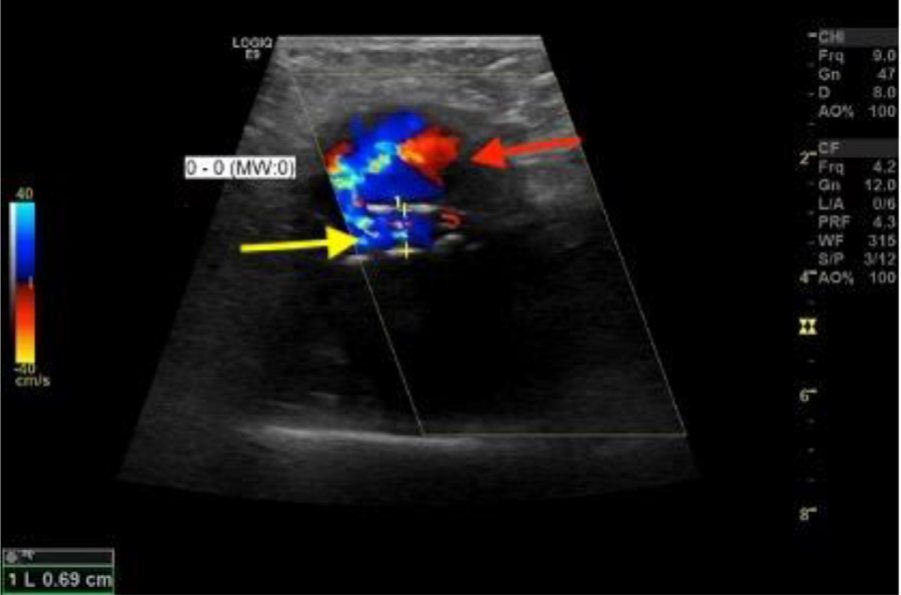
Duplex- Ultrasonography of the popliteal artery. Legend 4: Turbulent flow out of the popliteal artery, in the lower part (yellow arrow) of the popliteal artery (0.69 cm) and above it the extraluminal blood flow exiting through the injured vessel wall forming the pseudoaneurysm (marked with red arrow).

Initially, an x-ray of the left knee joint was performed (Fig. 1). No bone fractures could be seen; however, in the lateral x-ray view, a backward-arched displacement of the calcified popliteal artery was obvious. Subsequently, duplex ultrasonography was performed, which showed a large hematoma with a diameter of 6.8 cm and a central turbulent flow (Fig. 2). The hematoma featured marginal thrombotic formations (Fig. 3) and was fed by the active turbulent flow out of the popliteal artery. The popliteal artery itself was heavily calcified and moderately enlarged (0.69 cm in diameter) (Fig. 4); distal to the hematoma it showed post-obstructive mono-phase flow in pulse mode. Based on these duplex ultrasonography findings, a pseudoaneurysm in the proximal part of the left popliteal artery was established as the working diagnosis.

**Fig. 5:**
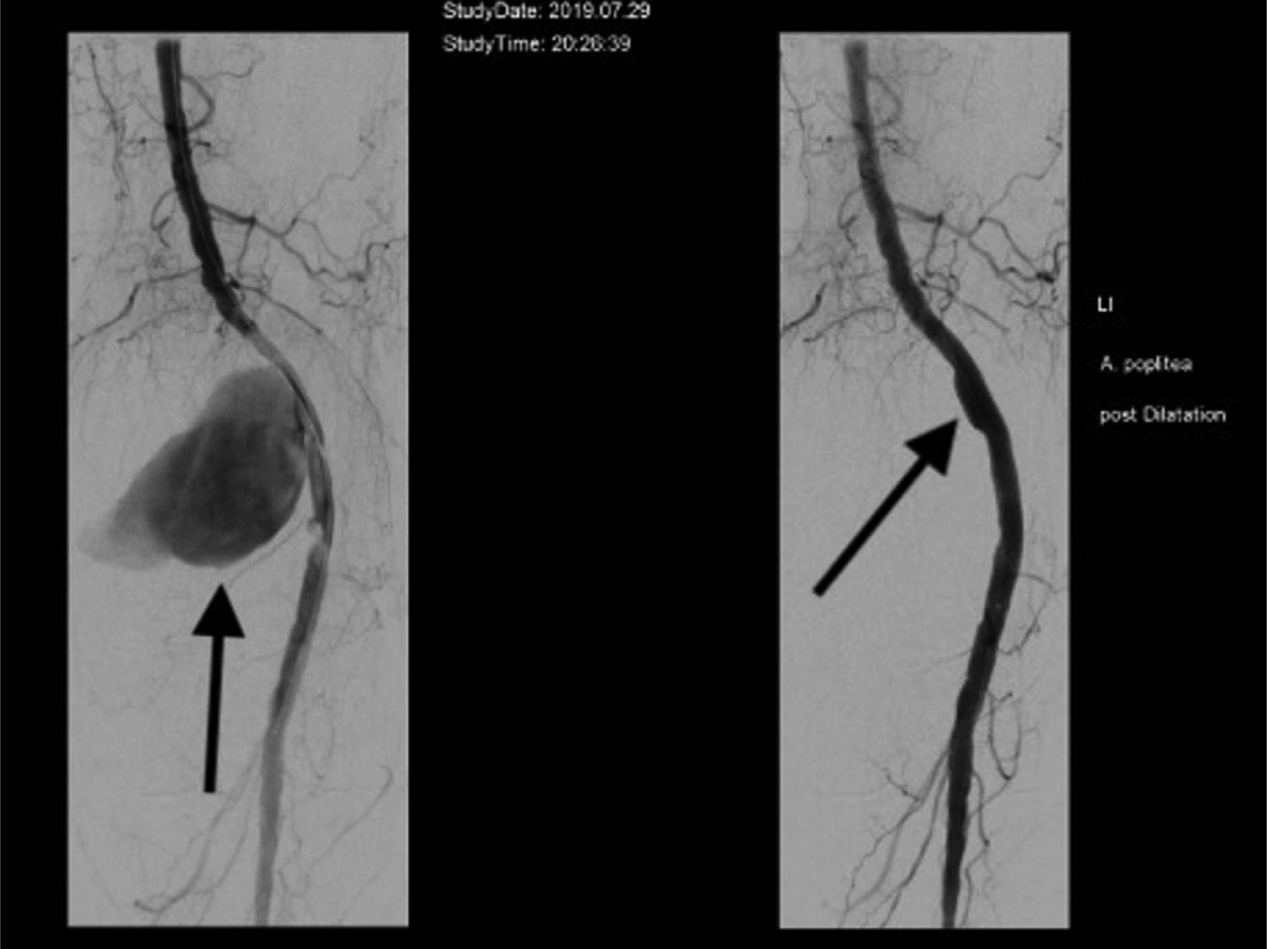
Digital subtraction angiography of the left popliteal artery. Legend 5: The angiography confirms a large aneurysm of the popliteal artery. On the left image marked with an arrow the perfused pseudoaneurysm. In the right picture, the arrow marks the place where the pseudoaneurysm could be closed by a covered stent.

After the diagnosis of an idiopathic pseudoaneurysm of the popliteal artery was established, a multidisciplinary case conference involving vascular surgeons and interventional radiologists was held to discuss the treatment options. The 2 potential approaches were open surgery and endovascular intervention. However, due to the patient's advanced age and multiple comorbidities, there was a high risk associated with general anesthesia. Therefore, the decision was made to proceed with a minimally invasive endovascular approach using local anesthesia. This approach would minimize the anesthetic risk while still effectively treating the pseudoaneurysm. A digital subtraction angiography (DSA) by antegrade femoral access with a 7 French sheath in local anesthesia was performed (Fig. 5). A covered Stent (Viabahn, 8 mm diameter, 100 mm length) was implanted into the left popliteal artery and thus the complete pseudoaneurysm was successfully closed (Fig. 5).

## Discussion

Injuries between the 2 outer layers of an artery may result in pseudoaneurysm [4,5].

With the number of endovascular procedures performed worldwide increasing, pseudoaneurysms mostly affect the common femoral arteries, as they are the regular access site for a large part of these interventions [2]. Compared to pseudoaneurysms of the common femoral artery, PPAs are rather uncommon and mostly associated with prior medical interventions or trauma [4,5].

In particular orthopedic [4,6] and vascular procedures [4,7], as well as acupuncture [4,[8], [9], [10], [11] in the region of the knee joint can lead to unintentional vascular damage resulting in pseudoaneurysm. Autoimmune rheumatic diseases [4,12] or local infections [13] are seldom considered to cause PPAs.

In contrast, a true aneurysm is a focal dilatation of all 3 layers of the wall of an artery, with the largest diameter being more than 50% of the normal. These focal dilations classify as fusiform (diffuse dilation) or saccular (asymmetrical). The exact incidence of true popliteal artery aneurysm is not known, the estimated incidence is 7.39 per 100,000 people [15] with a male-to-female ratio of about 15:1.

In our case, the elderly multimorbid patient could not adequately provide information on her medical history due to her severely reduced state of health. However, in Austria, every medical report of the last 20 years is available on an electronic database. By searching the patient´s medical history in that database, prior surgery as a cause of the pseudoaneurysm could be excluded; a traumatic origin also didn´t seem likely in a bedridden patient. We, therefore, considered our patient´s pseudoaneurysm as idiopathic. The leading symptom of PPAs is a large pulsating mass in the fossa poplitea. Frequent accompanying symptoms such as pain, swelling of the lower leg, and limited leg extension movement might, however, lead to consideration of numerous differential diagnoses such as venous thromboembolism, Baker´s cyst, gonarthrosis, or bone fracture. Therefore further diagnostic imaging is essential to confirm the diagnosis of PPA [16]. After bone fracture being ruled out as a differential diagnosis using X-rays, an experienced clinician can perform duplex sonography to diagnose vascular expansion and to exclude possible differential diagnoses, such as venous thrombosis, at the same time. Nevertheless, arteriography by arterial puncture is considered the gold standard in the diagnosis of pseudoaneurysm of the arteries [17]. In contrast, sonography, MRI, or computed tomography are suitable for the diagnosis of a true aneurysm. Duplex sonography in particular provides excellent visualization of the disrupted vessel wall in a pseudoaneurysm with extraluminal blood flow and typical pendulum flow. As described above, in a true aneurysm the vessel wall is not perforated but focally dilated, often with a thrombotic rim and in any case without blood flow outside the vessel wall layers.

There are 2 main treatment approaches for PPA´s with comparable short and medium-term results: open surgery by vein interposition and endovascular intervention with the exclusion of the pseudoaneurysm with covered stents [18,19]. Endovascular intervention as a minimally invasive approach offers the advantage of avoiding the need for general anesthesia and was, therefore, the therapy of choice in our case. It is interesting to note that Mori et al. treated their case of asymptomatic popliteal pseudoaneurysm with surgical repair, while your case was treated with an endovascular approach [20]. This highlights the different treatment options available for pseudoaneurysms of the popliteal artery and the importance of tailoring treatment to the individual patient's needs and circumstances. In both cases, the goal was to effectively treat the pseudoaneurysm and prevent potential complications such as rupture or thrombosis.

In conclusion, our case report describes the successful diagnosis and treatment of an idiopathic popliteal pseudoaneurysm in a bedridden elderly patient using noninvasive diagnostic procedures and a minimally invasive endovascular approach. The interdisciplinary case conference with vascular surgeons and interventional radiologists allowed for a well-informed decision on the most suitable treatment approach, taking into consideration the patient's high anesthesiologic risk due to her multimorbidity. This case highlights the importance of prompt and accurate diagnosis of rare vascular pathologies, as well as the potential benefits of minimally invasive endovascular intervention in selected cases.

## Ethical approval

For this type of study formal consent is not required.

## Authors' contributions

MR, PR designed the study; PR collected data; AR, HBC, PR analyzed and interpreted data; MR, PR wrote the manuscript; MR, HBC, AR, PR validated, reviewed, and edited the manuscript.

## Patient consent

Informed consent for publication was obtained from the patient.
